# Investigating the temporal dynamics of suspended sediment during flood events with ^7^Be and ^210^Pb_xs_ measurements in a drained lowland catchment

**DOI:** 10.1038/srep42099

**Published:** 2017-02-07

**Authors:** Marion Le Gall, Olivier Evrard, Anthony Foucher, J. Patrick Laceby, Sébastien Salvador-Blanes, Louis Manière, Irène Lefèvre, Olivier Cerdan, Sophie Ayrault

**Affiliations:** 1 Laboratoire des Sciences du Climat et de l’Environnement, UMR 8212 (CEA/CNRS/UVSQ), Université Paris-Saclay, Domaine du CNRS, Gif-sur-Yvette (France); 2 E.A 6293, GéoHydrosystèmes Continentaux (GéHCO), Université F. Rabelais de Tours, Faculté des Sciences et Techniques, Tours (France); 3 Département Risques et Prévention, Bureau de Recherches Géologiques et Minières, Orléans (France)

## Abstract

Soil erosion is recognized as one of the main processes of land degradation in agricultural areas. High suspended sediment loads, often generated from eroding agricultural landscapes, are known to degrade downstream environments. Accordingly, there is a need to understand soil erosion dynamics during flood events. Suspended sediment was therefore sampled in the river network and at tile drain outlets during five flood events in a lowland drained catchment in France. Source and sediment fallout radionuclide concentrations (^7^Be, ^210^Pb_xs_) were measured to quantify both the fraction of recently eroded particles transported during flood events and their residence time. Results indicate that the mean fraction of recently eroded sediment, estimated for the entire Louroux catchment, increased from 45 ± 20% to 80 ± 20% between December 2013 and February 2014, and from 65 ± 20% to 80 ± 20% in January 2016. These results demonstrate an initial flush of sediment previously accumulated in the river channel before the increasing supply of sediment recently eroded from the hillslopes during subsequent events. This research highlights the utility of coupling continuous river monitoring and fallout radionuclide measurements to increase our understanding of sediment dynamics and improve the management of soil and water resources in agricultural catchments.

Intensive agricultural activities often result in an increase of erosion and fine sediment supply to river networks^1^,^2^. An excessive supply of fine sediment to water bodies may result in the sedimentation and the siltation of channels, reservoirs and estuaries^3^. Furthermore, fine sediment may transport contaminants such as metals, organic compounds and nutrients or radionuclides^4^,^5^,^6^. These substances are mainly associated with the <63 μm fraction of particles and contribute to reductions in water quality^1^.

Currently, there is a need to improve our understanding of sediment generation, transport and deposition processes in agricultural catchments in order to design efficient sediment management measures and reduce the fine particle supply to water bodies. This is particularly important in cultivated catchments of Northwestern Europe^7^,^8^ where the intensification of agriculture after World War II resulted in an increase of sediment yields^9^. Knowledge of erosion processes, sediment sources and dynamics is particularly limited for wetland catchments where tile drains were installed after 1945 to produce crops in these former cattle breeding areas^10^,^11^. Although several studies suggest that these drainage systems may increase the connectivity between cultivated hillslopes and the river network^12^,^13^, additional information is needed to characterize the nature and the transit times of the material transported through these systems.

Natural fallout radionuclides (^7^Be, ^210^Pb_xs_) with different half-lives were shown to be useful tracers to quantify sediment dynamics in river systems^14^,^15^,^16^. ^7^Be is a short-lived cosmogenic radionuclide (t_1/2_ = 53 days) generated in the stratosphere and the upper troposphere by cosmic ray spallation of nitrogen and oxygen^17^. In contrast, ^210^Pb_xs_ (t_1/2_ = 22.3 years) is a longer-lived radionuclide, which is a product of ^238^U (t_1/2_ = 4.5.10^9^ years) that decays into ^226^Ra (t_1/2_ = 1600 years) and ^222^Rn (t_1/2_ = 3.8 days), a gas which mainly remains in soils forming “supported” ^210^Pb. A fraction of the “supported” ^210^Pb escapes to the atmosphere, generating “unsupported” or “excess” ^210^Pb during its subsequent fallout. Both ^7^Be and ^210^Pb_xs_ are mainly supplied to the soil surface by precipitation. Once they reach the soil surface, these radionuclides are strongly bound to fine particles^18^.

Measuring these radionuclides in both precipitation (i.e. fallout) and sediment provides a way to discriminate between particles that have been recently eroded on hillslopes, enriched in ^7^Be, and material re-suspended from the channel bed, depleted in this radioisotope^14^,^19^,^20^. A further development of this method consists in calculating the ratio of both radioisotopes to determine percentages of recently eroded sediment and the residence times of sediment in river networks^19^,^20^,^21^,^22^,^23^,^24^. Following this approach, recently eroded sediment is considered to have been tagged with ^7^Be supplied by the last rainfall events (i.e. the corresponding residence time in the river network should not exceed 50 days), whereas ‘old’ sediment is depleted in ^7^Be because it deposited in the channel during the previous months and was resuspended during the investigated event (i.e the corresponding residence time in the river network generally exceeds 100 days)^20^,^23^. A critique of this approach suggested that the ^7^Be activity of sediment was controlled by the source of sediment and that subsurface material should be theoretically sheltered from atmospheric fallout^25^. One way to address this critique is to apply this method in catchments where sediment is almost exclusively derived from surface sources exposed to atmospheric fallout. Another challenge associated with this method was that recently mobilized sediment is characterized by a ^7^Be/^210^Pb_xs_ ratio similar to that of rainfall. To address this challenge, the analysis of sediment collected in ephemeral flow occurring on hillslopes in the catchment was recommended to characterize the signature of recent sediment sources^21^,^26^.

In this study, suspended sediment dynamics were investigated in the Louroux catchment (France), a small agricultural catchment (25 km^2^) representative of lowland drained areas of Northwestern Europe. Natural fallout radionuclides (^7^Be, ^210^Pb_xs_) were analyzed in overland flow and sediment samples collected at seven locations in the catchment during the floods that occurred in the 2013-2014 and the 2015-2016 winter seasons. Previous research examining ^137^Cs activities in material transiting the Louroux River indicated that sediment transported during flood events in this catchment almost entirely originated from surface sources (~99%)^13^. The dominance of surface sources exposed to atmospheric fallout in this catchment justifies the application of this method while addressing recent criticisms of this technique^25^.

## Results

### Flood characteristics

**Figure 1:**
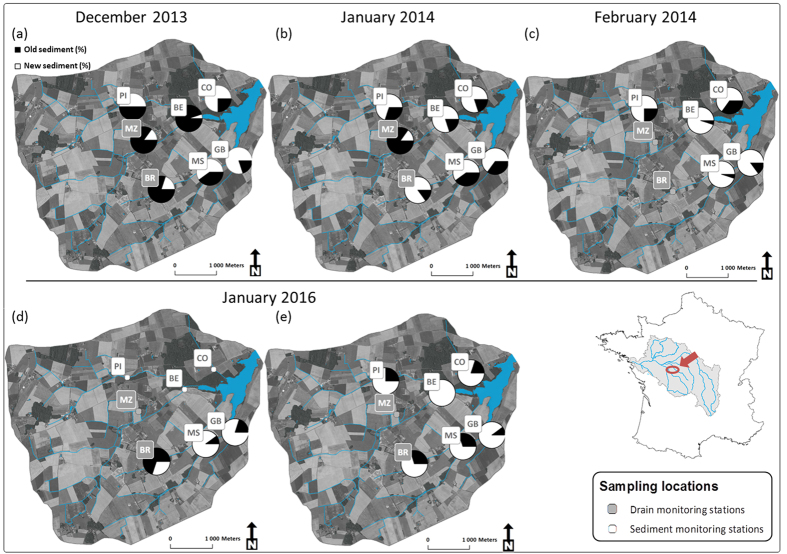
Evolution of the fraction of recently eroded sediment (%) during the five monitored floods. These original maps were created from data available from the BD Carthage database (https://www.data.gouv.fr/fr/datasets/bd-carthage-onm/) and from the IGN database (https://www.data.gouv.fr/fr/datasets/bd-carthage-onm/) using ArcGIS 10.2.1 software (http://www.esri.com/software/arcgis).

Hydrosedimentary parameters were recorded at seven locations in the Louroux catchment, including five stations installed on the main streams draining to the Louroux pond located at the outlet: Conteraye (CO), Picarderie (PI), Beaulieu (BE) on the northern tributaries of the pond, and Masniers (MS) and Grand Bray (GB) stations on the southern tributary. Furthermore, two additional stations monitored tile drain outlets: Mazère (MZ) (northern tributary) and Brépinière (BR) (southern tributary) stations (Fig. 1).

**Figure 2:**
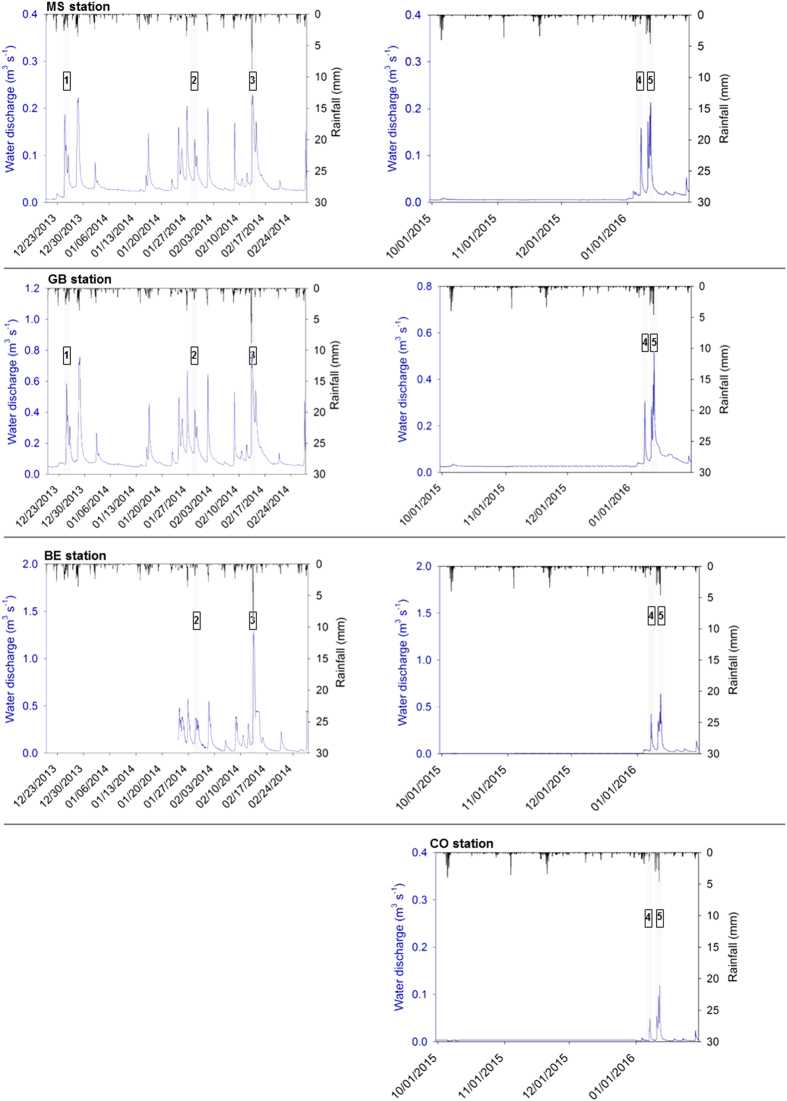
Evolution of water discharge (m^3^ s^−1^) (measured for the available MS, GB, BE and CO monitoring stations) and precipitation (mm) during the December 2013 to February 2014 (1,2,3) and the January 2016 (4,5) periods. The five monitored flood events (1, 2, 3, 4 and 5) are shown in grey. These original graphs were created using SigmaPlot 12.5 software (http://www.sigmaplot.co.uk/products/sigmaplot/produpdates/prod-updates18.php).

**Figure 3:**
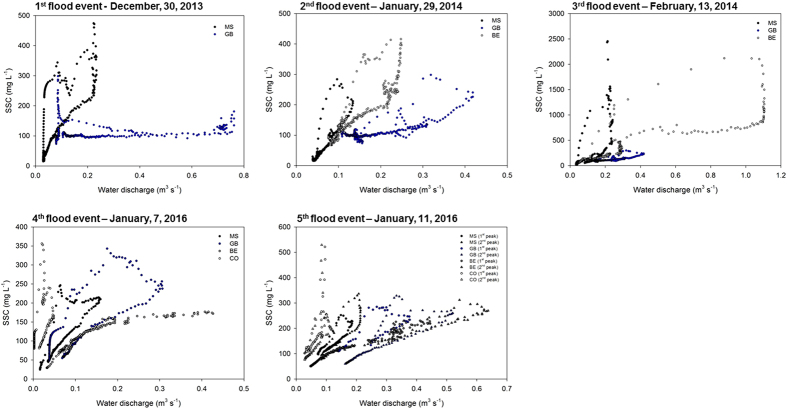
Water discharge and suspended sediment concentrations (SSC) for the five monitored flood events at the Masniers (MS), Grand Bray (GB), Beaulieu (BE) and Conteraye (CO) stations. These original graphs were created using SigmaPlot 12.5 software (http://www.sigmaplot.co.uk/products/sigmaplot/produpdates/prod-updates18.php).

**Figure 4:**
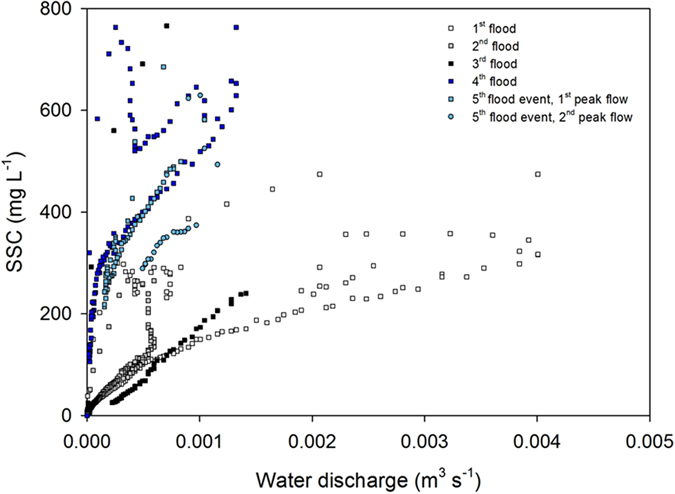
Water discharge and suspended sediment concentrations (SSC) for the five monitored flood event at the tile drain outlet of the Brépinière (BR) station. This original graph was created using SigmaPlot 12.5 software (http://www.sigmaplot.co.uk/products/sigmaplot/produpdates/prod-updates18.php).

In 2013-2014, the two first floods of the winter season were generated by long-lasting low-intensity rainfall (Fig. 2) and they were characterized by similar suspended sediment concentrations (Table S1). On December 30, 2013 (1^st^ flood) and January, 29, 2014 (2^nd^ flood), discharge was the highest at the GB monitoring station (0.76 m^3^ s^−1^ and 0.42 m^3^ s^−1^ respectively). During the 1^st^ flood, sediment export was the highest at the GB station with a flux of 22 t, while during the 2^nd^ flood, the sediment export reached a maximal value of 8 t at the BE station (Table S1). On February, 13, 2014, the 3^rd^ flood event was more intense (Fig. 2) and characterized by the highest water discharge recorded during the study period, with a maximal value of 1.1 m^3^ s^−1^ measured at the BE station, corresponding to a maximal export of sediment of 66 t. Due to the high cumulative precipitation recorded during this period, soils were saturated and the additional rainfall generated erosion and sediment transport with higher suspended sediment concentrations during the 3^rd^ event relative to the first two events investigated in 2013 (Table S1). In 2016, the two flood events were characterized by similar discharges, which were the highest at the BE station with respective maximal values of 0.43 and 0.64 m^3^ s^−1^ on January 7 (4^th^ flood) and January 11 (5^th^ flood), corresponding to respective maximal sediment fluxes of 3.3 and 9.5 t (Table S1). During the five flood events monitored, clockwise hysteresis loops were observed with higher suspended sediment concentrations during the rising limb of the flood compared to the falling limb, for a given value of water discharge (Fig. 3 and Fig. 4).

### Radionuclide activities (^7^Be and ^210^Pb_xs_) in suspended sediment

Radionuclide activities were measured in sediment collected in overland flow occurring in an ephemeral rill to characterize the recently eroded sediment source. For the 1^st^ flood event, ^7^Be and ^210^Pb_xs_ activities reached respective values of 420 ± 10 Bq kg^−1^ and 50 ± 10 Bq kg^−1^. During the 2^nd^ and 3^rd^ flood events, activities were lower with respective ^7^Be activities of 120 ± 10 Bq kg^−1^ and 165 ± 5 Bq kg^−1^ and ^210^Pb_xs_ activities of 10 ± 5 Bq kg^−1^ and 25 ± 5 Bq kg^−1^ (Table S2). In 2016, ^7^Be and ^210^Pb_xs_ activities ranged between 175 ± 5 Bq kg^−1^ and 30 ± 5 Bq kg^−1^ respectively. These activities could only be measured for the 4^th^ flood event in 2016 (Table S3).

For suspended sediment collected in 2013-2014, ^7^Be activities were more variable than ^210^Pb_xs_ activities which remained of the same order of magnitude during the three floods, with values that did not exceed 20 ± 5 Bq kg^−1^. The two first floods exhibited large variations in ^7^Be concentrations, ranging between 10 ± 20 and 130 ± 5 Bq kg^−1^. In contrast, the third flood exhibited lower variations of ^7^Be activities, with values between 100 ± 5 and 130 ± 10 Bq kg^−1^ (Table S2).

For suspended sediment sampled during the 4^th^ flood in 2016, ^7^Be activities ranged between 60 ± 10 and 135 ± 10 Bq kg^1^ and ^210^Pb_xs_ activities varied between 20 ± 5 and 40 ± 10 Bq kg^−1^. For the 5^th^ flood, ^7^Be activities were higher, with values ranging between 120 ± 15 and 415 ± 25 Bq kg^−1^ while ^210^Pb_xs_ activities remained in the same order of magnitude with values varying between 25 ± 5 and 70 ± 10 Bq kg^−1^. During this 5^th^ flood, sediment transported in ephemeral flow could unfortunately not be sampled for logistical reasons. Consequently, the highest ^7^Be activity of 415 ± 25 Bq kg^−1^ measured in suspended sediment collected at the BE station was used to characterize the signature of recently eroded particles for this event (Table S3).

### Evolution of the fraction of recently eroded particles during the five flood events

^7^Be/^210^Pb_xs_ ratios were calculated and used to estimate the fraction of recently eroded sediment and the mean residence time of particles for each event and each station (Tables S2 and S3). To address the fact that ^7^Be activities may differ in rainfall and in recently eroded sediment, ^7^Be activities were measured in overland flow and used as the recently eroded sediment source signature.

For the 2013-2014 winter season, the 1^st^ flood was characterized by similar fractions of recently eroded particles in both northern (CO, PI, MZ and BE stations) and southern (BR, MS and GB stations) tributaries (Fig. 1a). An increase in the fraction of recently eroded particles was observed in downstream direction from 20 ± 5% at BR to 80 ± 50% at GB stations in the southern tributary of the catchment. Variations were higher during the 2^nd^ flood event with values ranging between 15 ± 15% (MZ) and 85 ± 50% (BR) (Fig. 1b). The 3^rd^ flood was characterized in the northern tributaries by similar fractions of recently eroded sediment compared to the 2^nd^ flood event with values ranging between 65 ± 15% (CO) and 95 ± 25% (BE) while in the southern tributary high fractions were observed at the MS (95 ± 25%) and GB (85 ± 20%) stations (Fig. 1c) (Table S2).

For the 4^th^ flood, the fraction of recently eroded sediment ranged between 30 ± 10% (BR) and 90 ± 20% (MS) (Fig. 1d) (Table S4). During the 5^th^ flood, recently eroded particles dominated at all monitoring stations with contributions ranging between 70 ± 15% (MS) and 70 ± 20% (BR) to 100 ± 25% (BE). The lowest contributions of recently eroded sediment were observed at the uppermost BR (70 ± 20%) and PI (75 ± 15%) stations whereas the highest contributions were observed downstream, at GB (90 ± 20%) and BE (100 ± 25%) stations (Fig. 1e, Table S4).

Mean proportions of recently eroded particles and particle residence times were calculated for each flood to facilitate inter-event comparison. In 2013–2014, the fraction of recently eroded particles increased during the succession of floods (mean values of 45 ± 20% for the 1^st^ flood, 65 ± 35% for the 2^nd^ flood, 85 ± 20% for the third flood). In 2016, proportions of recently eroded particles increased between both events (65 ± 20% for the 4^th^ flood; 80 ± 20% for the 5^th^ flood) (Table S5).

### Intra-event variations of the fraction of recently eroded particles during the 2016 flood events

**Figure 5:**
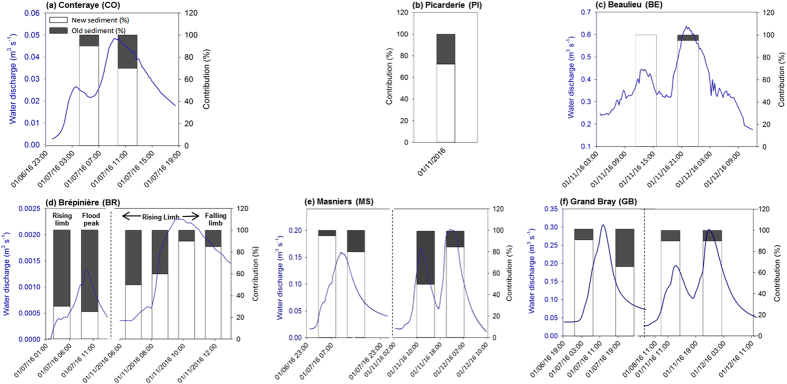
Variations of the fraction of recently eroded particles (%) during the two successive flood events in January, 2016 presented with the hydrographs (not available for the PI and BR stations). These original graphs were created using SigmaPlot 12.5 software (http://www.sigmaplot.co.uk/products/sigmaplot/produpdates/prod-updates18.php).

For the 4^th^ flood event, steady contributions were observed during the rising stage and the flood peak at the BR station (30 ± 5% and 25 ± 10%) and during both the rising and falling limbs at the MS station (95 ± 25% and 80 ± 20%) (Fig. 5d,e). For suspended sediment collected at the GB station, the highest contribution of recently eroded particles was observed during the rising limb (90 ± 40%) compared to the falling limb (65 ± 25%) (Fig. 5f, Table S3).

For the 5^th^ event, contributions of recently eroded particles in the northern tributaries remained comparable between the first (90 ± 15% at CO station and 100 ± 25% at BE station) and second flood peaks (70 ± 15% and 95 ± 25%, respectively) (Fig. 5a,c). In the southern tributary, at the tile drain outlet of BR, an increase of the fraction of recently eroded particles was observed with contributions increasing from 50 ± 15% to 90 ± 20% during the rising limb of the flood (Fig. 5d). During the falling limb, a slight decrease was observed with a mean contribution of recently eroded particles of 85 ± 25%. At the MS station, the highest contribution of recently eroded particles was observed during the second flood peak (85 ± 20% compared to 50 ± 10%) while steady contributions of surface sediment were observed at the GB station (90 ± 20% for the first and second flood peaks) (Fig. 5e,f).

When intra-event variations in sediment dynamics are investigated, the interpretation of proportional source data is particularly instructive^27^. Accordingly, intra-event contributions of recently eroded and remobilized particles were weighted using mass fluxes (Fig. S1). For the 4^th^ event, the sediment export and contributions of recently eroded particles were similar during the rising and falling limbs at the MS station (Table S3). At the GB station, although the contribution of recently eroded particles decreased between the rising and falling limbs of the flood, sediment fluxes strongly increased (Table S3). For the 5^th^ event, the sediment export and contributions of recently eroded particles increased between the first and the second flood peaks at the MS station. At the GB station, sediment fluxes decreased although the fraction of recently eroded particles increased (Fig. S1, Table S3).

## Discussion

### Hydro-sedimentary processes and sediment sources

Hysteresis patterns have been widely used to investigate geomorphic processes occurring in catchments and identify the spatial sources delivering sediment^28^,^29^. In the Louroux catchment, clockwise hysteresis loops were observed for the five monitored flood events, suggesting that after a rapid export from the river channel, the suspended sediment stock may be exhausted before the end of the flood. These hysteresis loops are generally attributed to a depletion of available sediment before the flood peak discharge is reached^30^ and were shown to be the dominant hysteresis type in well-drained agricultural catchments^31^. Furthermore, clockwise hysteresis patterns were shown to be related to the contribution of proximal sediment sources^32^. Accordingly, clockwise hysteresis patterns observed in the Louroux catchment suggest that suspended sediment originates from proximal sources.

### Temporal dynamics of suspended sediment within floods and during successive flood events

The use of ^7^Be to investigate sediment dynamics relies on the assumption that no pre-existing ^7^Be is found across the area of investigation. This condition is reached after a long dry period when the inventory of pre-existing ^7^Be decreases through radioactive decay and when material has not been supplied to the river because rainfall erosivity or soil saturation with water remained too low. The first monitored flood event occurred six weeks after the first event of the winter season (November 11, 2013). Sediment fluxes were 2 to 45% lower than those exported during the first monitored flood. Furthermore, ^7^Be activities were measured in two sediment samples collected after this first winter flood on November 19, 2013 and ^7^Be activities remained below the detection limits suggesting that no pre-existing ^7^Be was stored in the river system. For the 2015-2016 winter season, no flood event was recorded before January 2016 (Fig. 2). Accordingly, ^7^Be may be used to document short-term sediment dynamics in the Louroux catchment.

The fraction of recently eroded particles increased during the successive flood events monitored during 2013-2014 and 2015-2016 winter seasons. In 2013-2014, the erosive and monitored flood (December 2013) generated a large export of sediment accumulated in the riverbed during the previous months, especially in areas where the fraction of recently eroded particles was the lowest (MZ, BR and BE stations). Despite a higher mean contribution of recently eroded particles during the second flood (January 2014), the southern GB station was characterized by the transit of a lower fraction of recently eroded particles, suggesting the accumulation of sediment in this area. Finally, the third flood (February 2014), which was more intense, mainly exported recently eroded particles (mean fraction of recently eroded particles was estimated to be 80 ± 20%). Accordingly, the two first monitored floods partially exported sediment accumulated during the previous dry period. The first monitored flood mainly exported older sediment compared to the third larger event that mainly transported sediment directly eroded from hillslopes and flushed the previously deposited sediment, which is illustrated by the highest estimated contributions of recently eroded sediment, close to 100%. In 2016, the same trend was observed in the southern tributary of the catchment, with an increase of the fraction of recently eroded particles between both events. A similar sediment turn-over cycle likely explains the variations observed in 2016. The analysis of several samples collected during the two floods investigated in 2016 confirms the validity of this interpretation. The weighted contributions estimated using sediment export data also corroborate these variations with increasing sediment exports throughout a flood event reducing the impact of a potential decrease in the contribution of recently eroded sediment.

### Spatial variations of suspended sediment during a flood event

Overall, an increase of the fraction of recently eroded particles was observed in the downstream direction during each event. During each flood, the progressive depletion of recently eroded particles observed at the upper stations before propagating to the lower stations indicates that sediment previously accumulated in the river channel sections is progressively exported to lower reaches of the river network. In December 2013 and January 2016, low contributions of recently eroded particles were estimated at the tile drain outlets of the BR and MZ stations, suggesting that drains transported ^7^Be-dead sediment accumulated during the previous wet season. In the Louroux catchment, flow from the drains mainly takes place between November and April, when low-intensity and long-lasting rainfall occur, leading to soil saturation. Then, from May to September, these drains remain dry and it is very likely that the contribution of the drain tiles remains very low during this period. This is consistent with the results of the first monitored erosive floods during which particles depleted in ^7^Be corresponding to sediment previously deposited in the riverbed and in the drains were exported. It would be useful to sample sediments from larger events to investigate whether there is some threshold where the drainage system starts to export sediment recently eroded from the surface soils.

### The use of ^7^Be and ^210^Pb_xs_ to investigate suspended sediment dynamics

Previous modelling results based on ^137^Cs measurements showed that suspended sediment transported in the Louroux pond tributaries during flood events were almost entirely supplied by surface sources (99 ± 0.5%)^13^. Although the number of studies using ^7^Be/^210^Pb_xs_ ratios to estimate the fraction and/or the age of suspended sediment increased recently^15^,^19^,^21^,^22^, it is difficult to compare their results to those obtained in the Louroux catchment. Indeed, climate and environmental conditions as well as spatial and temporal scales covered in the published studies are strongly variable. Matisoff *et al*.^20^ investigated sediment transport in three small US agricultural catchments (< 100 km^2^) and estimated recently eroded contributions comprised between 36 ± 6% to 55 ± 21%. Limited variations were observed in this study compared to the Louroux catchment (0–100%), highlighting its high reactivity, which is similar to other US agricultural^33^ (5–100%) and mountainous^22^ (12–96%) catchments. Although Walling (2012) questioned the use of ^7^Be in sediment fingerprinting research, our study demonstrates that if particles predominantly originate from surface sources, the sampling of sediment directly in an ephemeral rill during flood events, rather than the sampling of rainfall, may improve both the interpretation of ^7^Be/^210^Pb_xs_ ratios and the quantification of recently eroded particle contributions.

### Sediment transport in drained catchments and management implications

The progressive increase of recently eroded particles in the downstream direction, within a single flood and for a succession of floods, demonstrate the very rapid turn-over of sediment in this drained catchment. After a progressive remobilization of ^7^Be-dead material stored in the tile drains and the river channel, recent material tagged with ^7^Be is directly exported downstream. The material transiting the drains and the river network is supplied by surface proximal sources, as indicated by the observation of clockwise hystereses.

The high contribution of recently eroded particles suggests a high connectivity between the hillslopes and the river network. The dense drainage network of the Louroux catchment may act as a preferential pathway for these eroded particles. Sediment sources are controlled by the sequence of rainfall events and the total amount of rain generating floods^34^. Radionuclide measurements demonstrated that the first monitored erosive flood occurring at the beginning of the hydrological year mainly exported sediment accumulated in the tile drains and the river bed.

In this erosive catchment, specific management strategies must be implemented to reduce soil losses and sediment transport. To improve the understanding of suspended sediment sources and dynamics, more flood events should be investigated during an entire year. Indeed, their seasonal variability must be better understood to guide the implementation of efficient strategies to reduce sediment loads and limit sediment accumulation in the river channels. Further research should investigate intra-event variability using sediment fluxes data to weight the contributions of sediment sources. Attention should also focus on the drainage network which increases the connectivity between the hillslopes and the river network. In the future, complementary approaches including the analysis of clay mineralogy, developed for investigating clay lessivage in soil profiles^35^,^36^, could improve the discrimination between the particles transiting the tile drains and those supplied by ephemeral rills occurring on hillslopes.

## Methods

### Study site

The Louroux catchment (25 km^2^) is a small agricultural area located in the Loire River basin (France). The climate is temperate oceanic, with a mean annual rainfall of 684 mm (between 1971 and 2000 for the nearby city of Tours; Météo France, 2015^37^). The catchment is characterized by a flat topography (mean slope of 0.4%), and cropland is the main land use (78%) although slight variations may be observed between subcatchments (Table 1) (Corine Land Cover 2006 data). The five main streams draining the Louroux pond, located at the outlet of the catchment, are equipped with monitoring stations (CO, PI, BE, MS, GB, Fig. 1) along with two additional stations located at tile drain outlets (MZ, BR, Fig. 1).

### Sampling

Hydro-sedimentary parameters (i.e. water level, turbidity) were continuously recorded at the seven monitoring stations equipped with automatic samplers. Suspended sediment concentrations were estimated based on turbidity measurements. Turbidity was calibrated to provide suspended sediment concentrations using a rating curve (CO station, R^2^ = 0.936, n = 53; BE station, R^2^ = 0.946, n = 94; MS station, R^2^ = 0.945, n = 97; GB station, R^2^ = 0.928, n = 58; BR station, R^2^ = 0.946, n = 67) constructed with the analysis of samples collected manually during low flow periods and flood events. Rainfall intensity was monitored at the outlet of the catchment with an automatic weather station.

Three flood events were investigated in 2013-2014 (December 30, 2013, January 29, 2014 and February 13, 2014) and two successive flood events were studied in 2015-2016 (January 7 and 11, 2016). As ^7^Be activities in rainfall may not be representative of the corresponding soil/sediment labelling, overland flow was collected in an ephemeral rill located close to the CO station during each flood event to characterize fallout radionuclide in potential source sediment (except for the January 11, 2016 event). For each flood event, a single bulk integrated sample was collected in the rill. To further estimate the proportion of recently eroded sediment and the residence time of particles transiting the rivers, we assumed that no spatial variability in radionuclide activity occurred across this small catchment (25 km^2^). This hypothesis is reasonable given the low spatial variations observed in ^7^Be fallout at various locations across another catchment of similar size (22.4 km^2^)^38^ Samples (n = 39) were collected to characterize the signature of sediment transiting the river, including stream (n = 22) and tile drain outlet (n = 17) samples, and compare it to that of source sediment.

In 2013-2014, during the two first floods, river water samples were collected at all stations whereas during the third flood, samples were only collected at four river stations (sampling was not possible at Conteraye and tile drain stations). In 2015-2016, sub-samples of suspended sediment were collected during the rising and the falling limb of the hydrograph at three monitoring stations (GB, MS, BR) for the first flood and at six monitoring stations during the second flood (i.e. all stations except MZ station) to characterize intra-event variations of sediment signatures.

### Sample processing and analysis

#### Sample preparation

Sediment samples were oven-dried at 40 °C for 48 h^13^. Dry sediment (between 2 and 10 g) was then packed in containers and sealed airtight.

#### Calculation of sediment fluxes

For each station and monitored flood event, sediment fluxes were estimated using water discharge and suspended sediment concentration data recorded every 15 minutes.

#### Radionuclide measurements

The ^7^Be (477.6 keV) and ^210^Pb (46.5 keV) activities were determined by gamma spectrometry using low background N and P type GeHP detectors at the Laboratoire des Sciences du Climat et de l’Environnement and the Laboratoire Souterrain de Modane. ^210^Pb_xs_ activities were calculated by subtracting the supported activity (determined using two ^226^Ra daughters, ^214^Pb (average count number at 295.2 and 351.9 keV) and ^214^Bi (609.3 keV)) from the total ^210^Pb activity. Measured activities were decay-corrected to the sampling date. Associated uncertainties correspond to 2σ-errors for ^7^Be and ^210^Pb_xs_ measurements^21^. Samples were measured within 50 days to ensure that ^7^Be was detected.

#### Calculation of the contribution of recently eroded sediment

The percentage of recently eroded particles and the residence time of particles transiting the river network were calculating by using Eq. (1) and (2)^20^:


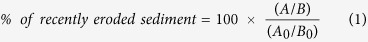



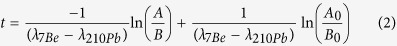


where λ_7Be_ and λ_210Pb_ are the decay constants of ^7^Be and ^210^Pb (day^−1^), A and B are ^7^Be and ^210^Pb_xs_ activities (Bq kg^−1^) in the suspended particulate matter, A_0_ and B_0_ are the ^7^Be and ^210^Pb_xs_ activities in sediment collected in overland flow occurring in an ephemeral rill.

## Additional Information

**How to cite this article**: Le Gall, M. *et al*. Investigating the temporal dynamics of suspended sediment during flood events with ^7^Be and ^210^Pb_xs_ measurements in a drained lowland catchment. *Sci. Rep.* **7**, 42099; doi: 10.1038/srep42099 (2017).

**Publisher's note:** Springer Nature remains neutral with regard to jurisdictional claims in published maps and institutional affiliations.

## Supplementary Material

Supplementary Information

## Figures and Tables

**Table 1:** Subcatchments’ surface area and relative land use distribution (%).

	Area (km^2^)	Cropland area (%)	Grassland/woodland area (%)	Urban area (%)
CO station	2.03	45	52	3
PI station	5.91	55	36	9
BE station	5.91	85	12	3
MS station	1.96	86	5	9
GB station	5.04	88	8	4
