# Supplementary material for: Investigating the temporal dynamics of suspended sediment during flood events with 7Be and 210Pbxs measurements in a drained lowland catchment
Source: Sci Rep. 2017 Feb 7;7:42099. doi: 10.1038/srep42099 (PMC5294465; doi:10.1038/srep42099)
Supplement: Supplementary Information [file srep42099-s1.pdf]

# Investigating the temporal dynamics of suspended sediment during flood events with $^{7}Be$ and $^{210}Pb_{xs}$ measurements in a drained lowland catchment

Marion Le Gall, Olivier Evrard, Anthony Foucher, J. Patrick Laceby, Sébastien Salvador-Blanes, Louis Manière, Irène Lefèvre, Olivier Cerdan, Sophie Ayrault

## Supplementary Information

**Table S1. Peak flow ($m^3 s^{-1}$), suspended sediment concentration measured for the peak flow (SSC in mg $L^{-1}$) and sediment export (ton) estimated for each monitoring station during the five flood events.**

| | Peak flow ($m^3 s^{-1}$) | Corresponding SSC (mg $L^{-1}$) | Sediment export (t) |
|---|---|---|---|
| **Suspended sediment - 1st flood event (12/30/2013)** | | | |
| Beaulieu (BE) | - | - | 0.7 |
| Masniers (MS) | 0.23 | 470 | 4.4 |
| Grand Bray (GB) | 0.76 | 510 | 22.0 |
| Brépinière (BR) | 0.004 | 345 | 0.03 |
| **Suspended sediment - 2nd flood event (01/29/2014)** | | | |
| Beaulieu (BE) | 0.25 | 415 | 8.0 |
| Masniers (MS) | 0.13 | 217 | 1.2 |
| Grand Bray (GB) | 0.42 | 240 | 6.1 |
| Brépinière (BR) | 0.0006 | 165 | 0.004 |
| **Suspended sediment - 3rd flood event (02/13/2014)** | | | |
| Beaulieu (BE) | 1.1 | 1500 | 66.0 |
| Masniers (MS) | 0.24 | 900 | 10.3 |
| Grand Bray (GB) | 0.79 | 1135 | 32.6 |
| Brépinière (BR) | 0.001 | 240 | 0.001 |
| **Suspended sediment - 4th flood event (07/01/2016)** | | | |
| Conteraye (CO) | 0.05 | 185 | 0.3 |
| Beaulieu (BE) | 0.43 | 170 | 3.3 |
| Masniers (MS) | 0.16 | 215 | 1.2 |
| Grand Bray (GB) | 0.31 | 255 | 3.1 |
| Brépinière (BR) | 0.001 | 660 | 0.02 |
| **Suspended sediment - 5th flood event (11/01/2016)** | | | |
| Conteraye (CO) | 0.12 | 195 | 1.3 |
| Beaulieu (BE) | 0.64 | 270 | 9.5 |
| Masniers (MS) | 0.21 | 335 | 2.7 |
| Grand Bray (GB) | 0.52 | 260 | 6.5 |
| Brépinière (BR) | 0.001 | 685 | 0.01 |

**Table S2. Fallout radionuclide activities (Bq kg$^{-1}$), $^{7}Be/^{210}Pb_{xs}$ ratios in suspended sediment and overland flow samples, residence time of particles (days), and fraction of recently eroded sediment for 2013-2014 flood events.**

| Monitoring station | $^{210}Pb_{xs}$ (Bq kg$^{-1}$) | $^{7}Be$ (Bq kg$^{-1}$) | $^{7}Be/^{210}Pb_{xs}$ | Residence time (days) | % of recently eroded sediment |
|---|---|---|---|---|---|
| **Suspended sediment - 1$^{st}$ flood event (12/30/2013)** | | | | | |
| Conteraye (CO) | 10 ± 5 | 60 ± 10 | 6.0 ± 1.8 | 25 ± 20 | 75 ± 25 |
| Picarderie (PI) | 10 ± 10 | 40 ± 10 | 4.0 ± 2.3 | 55 ± 40 | 50 ± 30 |
| Beaulieu (BE) | 20 ± 5 | 10 ± 20 | 0.5 ± 0.4 | 215 ± 60 | 5 ± 5 |
| Masniers (MS) | 10 ± 5 | 50 ± 5 | 5.0 ± 1.4 | 40 ± 20 | 60 ± 20 |
| Grand Bray (GB) | 10 ± 10 | 65 ± 10 | 6.5 ± 3.6 | 20 ± 40 | 80 ± 50 |
| Mazère (MZ) | 10 ± 10 | 10 ± 20 | 1.0 ± 1.2 | 160 ± 85 | 15 ± 15 |
| Brépinière (BR) | 15 ± 5 | 20 ± 5 | 1.5 ± 0.5 | 140 ± 15 | 20 ± 5 |
| **Suspended sediment - 2$^{nd}$ flood event (01/29/2014)** | | | | | |
| Conteraye (CO) | 10 ± 5 | 95 ± 10 | 9.5 ± 2.7 | 18 ± 0.6 | 80 ± 45 |
| Picarderie (PI) | 10 ± 5 | 85 ± 10 | 8.5 ± 2.5 | 30 ± 1 | 70 ± 40 |
| Beaulieu (BE) | 10 ± 5 | 130 ± 5 | 11.0 ± 1.2 | 20 ± 1 | 80 ± 20 |
| Masniers (MS) | 10 ± 5 | 70 ± 5 | 7.0 ± 1.9 | 40 ± 0.5 | 60 ± 30 |
| Grand Bray (GB) | 10 ± 5 | 75 ± 5 | 7.5 ± 2.1 | 35 ± 0.3 | 65 ± 35 |
| Mazère (MZ) | 20 ± 5 | 35 ± 70 | 1.8 ± 1.4 | 150 ± 40 | 15 ± 15 |
| Brépinière (BR)° | 5 ± 5 | 100 ± 30 | 10.0 ± 3.5 | 14 ± 5 | 85 ± 50 |
| **Suspended sediment - 3$^{rd}$ flood event (02/13/2014)** | | | | | |
| Conteraye (CO) | 30 ± 5 | 130 ± 10 | 4.3 ± 0.5 | 35 ± 0.2 | 65 ± 15 |
| Picarderie (PI) | 20 ± 5 | 100 ± 5 | 5.0 ± 0.5 | 25 ± 1.5 | 75 ± 15 |
| Beaulieu (BE) | 20 ± 5 | 130 ± 10 | 8.9 ± 1.3 | 5 ± 3 | 95 ± 25 |
| Masniers (MS) | 20 ± 5 | 120 ± 5 | 6.0 ± 0.8 | 10 ± 2 | 95 ± 25 |
| Grand Bray (GB) | 25 ± 5 | 140 ± 10 | 5.6 ± 0.7 | 15 ± 1 | 85 ± 20 |
| **Overland flow in an ephemeral rill** | | | | | |
| 1$^{st}$ flood event (12/30/2013) | 50 ± 10 | 420 ± 10 | 8.1 ± 0.4 | 0 | 100 |
| 2$^{nd}$ flood event (01/29/2014) | 10 ± 5 | 120 ± 10 | 12 ± 3.3 | 0 | 100 |
| 3$^{rd}$ flood event (02/13/2014) | 25 ± 5 | 165 ± 5 | 6.7 ± 0.8 | 0 | 100 |

**Table S3. Fallout radionuclide activities (Bq kg$^{-1}$), $^{7}Be/^{210}Pb_{xs}$ ratios in suspended sediment, residence time of particles (days), fraction of recently eroded sediment for 2016 flood events, number of sub-samples regrouped to measure radionuclide activities in each sample and sediment export (t) corresponding to the duration of the sampling collection.**

| Monitoring station | $^{210}Pb_{xs}$ (Bq kg$^{-1}$) | $^{7}Be$ (Bq kg$^{-1}$) | $^{7}Be/^{210}Pb_{xs}$ | Residence time (days) | % of recently eroded sediment | Number of samples | Sediment export (t) corresponding to each sample |
|---|---|---|---|---|---|---|---|
| **Suspended sediment - 4th flood event (01/07/2016)** | | | | | | | |
| Masniers - rising limb | 25 ± 5 | 135 ± 10 | 5.4 ± 0.7 | 5 ± 5 | 95 ± 20 | 11 | 0.58 |
| Masniers - falling limb | 20 ± 5 | 95± 10 | 5.4 ± 0.8 | 15 ± 4 | 80 ± 20 | 13 | 0.64 |
| Grand Bray - rising limb | 20 ± *20* | 130 ± 15 | 5.2 ± 1.8 | 10 ± 20 | 90 ± 40 | 5 | 0.38 |
| Grand Bray - falling limb | 20 ± 10 | 75 ± 10 | 3.8 ± 1.1 | 35 ± 15 | 65 ± 25 | 6 | 2.00 |
| Brépinière - rising limb | 40 ± 10 | 70 ± 5 | 1.7 ± 0.3 | 95 ± 5 | 30 ± 5 | 13 | - |
| Brépinière – flood peak | 40 ± 10 | 60 ± 10 | 1.5 ± 0.3 | 105 ± 5 | 25 ± 10 | 10 | - |
| **Overland flow in an ephemeral rill** | | | | | | | |
| 4th flood event (01/07/2016) | 30 ± 5 | 175 ± 5 | 5.8 ± 0.5 | - | - | | |
| **Suspended sediment - 5th flood event (01/11/2016)** | | | | | | | |
| Conteraye - 1st flood peak | 50 ± 5 | 380 ± 10 | 7.6 ± 0.4 | 10 ± 5 | 90 ± 15 | 11 | 0.40 |
| Conteraye - 2nd flood peak | 70 ± 10 | 410 ± 20 | 5.9 ± 0.5 | 25 ± 5 | 70 ± 15 | 13 | 0.76 |
| Picarderie - 1st and 2nd flood peaks | 50 ± 5 | 310 ± 15 | 6.2 ± 0.4 | 25 ± 3 | 75 ± 15 | 24 | |
| Beaulieu - 1st flood peak | 50 ± 10 | 415 ± 25 | 8.3 ± 1.0 | 0 | 100 ± 25 | 8 | 1.57 |
| Beaulieu - 2nd flood peak | 40 ± 10 | 320 ± 10 | 8.0 ± 1.1 | 5 ± 1 | 95 ± 25 | 16 | 6.19 |
| Masniers - 1st flood peak | 50 ± 10 | 195 ± 15 | 3.9 ± 0.5 | 60 ± 1 | 50 ± 10 | 11 | 0.74 |
| Masniers - 2nd flood peak | 30 ± 5 | 215 ± 10 | 7.2 ± 0.7 | 10 ± 1 | 85 ± 20 | 13 | 1.59 |
| Grand Bray - 1st flood peak | 45 ± 10 | 335 ± 10 | 7.4 ± 0.8 | 10 ± 1 | 90 ± 20 | 12 | 1.78 |
| Grand Bray - 2nd flood peak | 25 ± 5 | 200 ± 5 | 7.9 ± 0.6 | 5 ± 3 | 90 ± 20 | 11 | 0.54 |
| Brépinière - rising limb 1 | 40 ± 10 | 170 ± 20 | 4.3 ± 0.7 | 50 ± 5 | 50 ± 15 | 9 | - |
| Brépinière - rising limb 2 | 25 ± 10 | 120 ± 15 | 4.8 ± 1.2 | 40 ± 10 | 60 ± 20 | 6 | - |
| Brépinière - rising limb 3 | 25 ± 5 | 180 ± 10 | 7.2 ± 0.8 | 10 ± 1 | 90 ± 20 | 4 | - |
| Brépinière - falling limb | 30 ± 10 | 210 ± 20 | 6.9 ± 1.4 | 15 ± 5 | 85 ± 25 | 5 | - |

**Table S4. Mean residence time of particles and fraction of recently eroded particles for 2016 flood events.**

| Monitoring station | Age of sediment (days) | % of "new" sediment |
|---|---|---|
| **Suspended sediment - 4th flood event (01/07/2016)** | | |
| Masniers | 10 ± 5 | 90 ± 20 |
| Grand Bray | 20 ± 20 | 80 ± 35 |
| Brépinière | 100 ± 5 | 30 ± 10 |
| **Suspended sediment - 5th flood event (01/11/2016)** | | |
| Conteraye | 20 ± 5 | 80 ± 15 |
| Picarderie | 20 ± 5 | 75 ± 15 |
| Beaulieu | 5 ± 5 | 100 ± 25 |
| Masniers | 35 ± 5 | 70 ± 15 |
| Grand Bray | 5 ± 5 | 90 ± 20 |
| Brépinière | 30 ± 5 | 70 ± 20 |

**Table S5. Mean fraction of recently eroded sediment and residence time of particles during the five flood events.**

| | 2013-2014 | | | 2016 | |
|---|---|---|---|---|---|
| | 1st flood (a) | 2nd flood (b) | 3rd flood (c) | 4th flood (d) | 5th flood (e) |
| **% of recently eroded sediment** | 45 ± 20% | 65 ± 35% | 85 ± 20% | 65 ± 20% | 80 ± 20% |
| **Mean residence time of particles** | 80 ± 40 days | 45 ± 10 days | 20 ± 5 days | 45 ± 10 days | 25 ± 5 days |

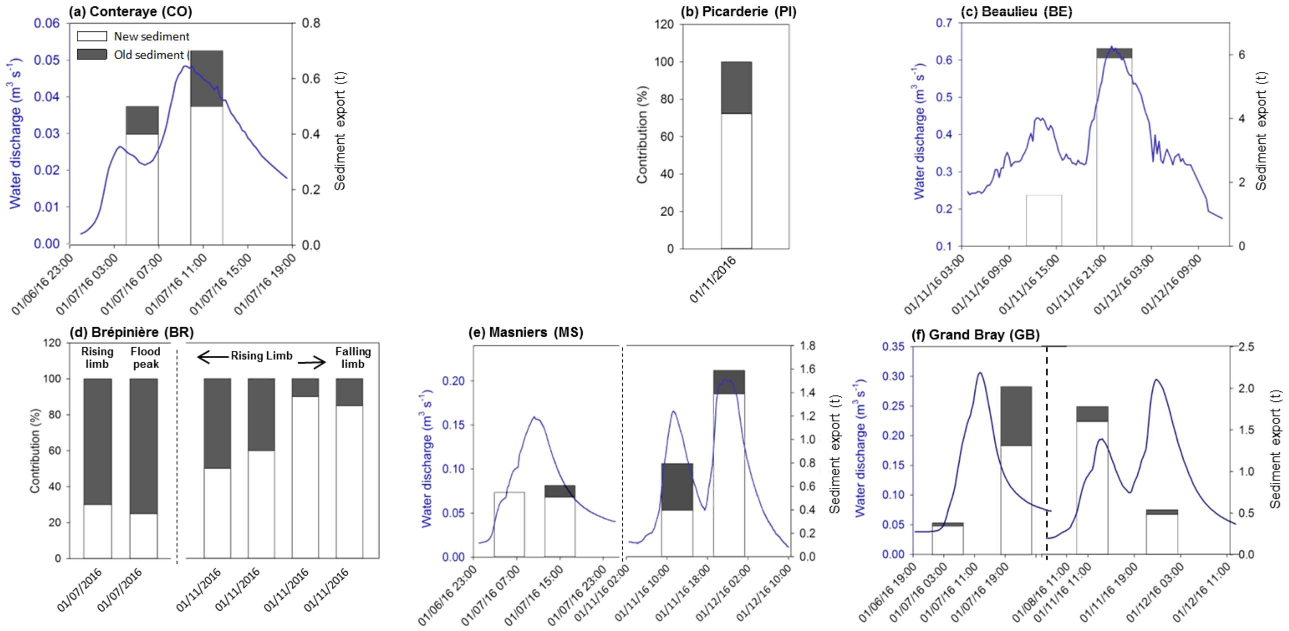


**Figure S1.** Variations of the contribution of recently eroded particles during the two successive flood events in January, 2016 presented with the hydrographs (not available for the PI and BR stations). For CO, BE, MS and GB stations, the contributions are weighted using sediment export data. These original graphs were created using SigmaPlot 12.5 software (http://www.sigmaplot.co.uk/products/sigmaplot/produpdates/prod-updates18.php).
